# Modulation of ferroptosis via YY1-SLC7A11 axis in hepatic ischemia-reperfusion injury pathogenesis: Ferroptosis modulation in hepatic IRI

**DOI:** 10.3724/abbs.2025093

**Published:** 2025-07-01

**Authors:** Shaochuang Wang, Baofei Jiang, Jun Cao, Ting Xu, Chengming Zhou, Xiangyou Yu, Yi Wang, Yu Xie, Lindong Ji, Guangrong Zhou, Hao Wen, Long Ma, Kun Wu

**Affiliations:** 1 Department of Hepatobiliary Surgery The Affiliated Huai’an No.1 People’s Hospital of Nanjing Medical University Huai’an 223300 China; 2Department of General Surgery Shanghai Tenth People’s Hospital Shanghai 200072 China; 3 State Key Laboratory of Pathogenesis Prevention and Treatment of High Incidence Diseases in Central Asia Xinjiang Medical University Urumqi 830054 China; 4 Hematology Research Laboratory The Affiliated Huai’an No.1 People’s Hospital of Nanjing Medical University Huai’an 223300 China; 5 Department of Critical Care Medicine The First Affiliated Hospital of Xinjiang Medical University Urumqi 830054 China; 6 Department of Gastrointestinal Surgery The Affiliated Huai’an No.1 People’s Hospital of Nanjing Medical University Huai’an 223300 China

**Keywords:** NEDD4L, YY1, SLC7A11, ferroptosis, hepatic ischemia-reperfusion injury, ubiquitination

## Abstract

YY1 is a crucial transcription factor and plays significant roles in biological processes. However, the mechanisms of YY1 action in ischemia-reperfusion injury and its regulatory role in ferroptosis have not been extensively studied. This study aims to elucidate the molecular mechanism by which NEDD4L-mediated degradation of YY1 through ubiquitination suppresses SLC7A11 transcription, leading to the promotion of cellular ferroptosis and exacerbation of hepatic ischemia-reperfusion injury (IRI), via the integration of multiple omics sequencing datasets. An IRI-I/R mouse model is established, followed by proteomic sequencing to identify proteins that are differentially expressed during IRI. The altered expression of YY1 is validated, and *in vivo* and *in vitro* experiments are used to assess its impact on IRI damage. The E3 ligase NEDD4L, which regulates YY1 ubiquitination, is identified and validated via the UbiBrowser 2.0 database. The ubiquitination types of YY1 and its sites are screened and confirmed through *in vitro* experiments. Transcriptional sequencing of YY1-overexpressing cell lines is conducted to analyze the involvement of the downstream transcription factor SLC7A11 in IRI, followed by validation of its regulatory role. The results show that YY1 is downregulated in liver tissues during IRI and is expressed primarily in liver cells. YY1 overexpression alleviates liver tissue and liver cell IRI both *in vitro* and *in vivo*. Upregulation of E3 ligase expression during IRI promotes the K63-linked ubiquitination of YY1 at the K339 site, leading to proteasomal degradation of YY1. RNA-seq analysis and experimental validation demonstrate that YY1 suppresses IRI-induced ferroptosis via the transcriptional regulation of downstream target genes. YY1 positively regulates SLC7A11 transcription, inhibits IRI-induced ferroptosis and ameliorates liver injury. In summary, the E3 ubiquitin ligase NEDD4L facilitates YY1 protein degradation through ubiquitination, suppressing the transcription of the ferroptosis inhibitor SLC7A11, thus promoting IRI-related ferroptosis and exacerbating liver injury.

## Introduction

Ischemia-reperfusion injury (IRI) is a significant pathological process present in diseases such as myocardial infarction, stroke, and organ transplantation [ 1– 3]. During the occurrence and development of IRI, cells are subjected to various types of damage, including oxidative stress, inflammatory reactions, and apoptosis. Recent studies have identified ferroptosis as a novel form of cell death that plays a crucial role in IRI, offering new insights into the study of this disease [ 4– 6]. Therefore, a thorough investigation of the molecular mechanisms of IRI is of paramount importance for unraveling the essence of disease occurrence and discovering new therapeutic strategies. 

YY1 is a protein that serves as a crucial transcription factor and plays significant roles in biological processes such as cell proliferation, differentiation, and death [ 7– 9]. However, the mechanisms of YY1 action in IRI and its regulatory role in ferroptosis have not been extensively studied [10]. Studies have indicated that downregulation of YY1 expression during IRI may be closely associated with the occurrence of ferroptosis [ 11– 13]. Therefore, a more detailed and in-depth exploration of the role of YY1 in IRI is crucial for understanding the pathogenesis of IRI. 

The E3 ubiquitin ligase NEDD4L is an important regulator of ubiquitination and is involved in the degradation and maintenance of cellular proteins through ubiquitination processes [ 14– 16]. Recent studies have revealed the critical role of NEDD4L in IRI, particularly its association with cellular ferroptosis [ 17, 18]. Specifically, YY1, a key transcription factor, is regulated by NEDD4L at the protein level, influencing the occurrence of ferroptosis during IRI. Therefore, investigating the specific mechanisms of NEDD4L in IRI not only aids in a deeper understanding of crucial aspects of the pathophysiology of IRI but also provides a new research perspective for the development of therapeutic strategies for this disease. 

Furthermore, this study focused on the downstream gene *SLC7A11* of YY1, which plays a pivotal role in ferroptosis [12]. The expression level of SLC7A11 is influenced by YY1 regulation, emphasizing the importance of studying the regulatory effect of YY1 on SLC7A11 [12]. By delving into the interaction between YY1 and SLC7A11, a better comprehension of the mechanism of action of YY1 in ferroptosis can be achieved, providing theoretical support for future research on IRI treatment [12]. 

The objective of this study was to elucidate how NEDD4L degrades YY1 through ubiquitination to inhibit the transcription of *SLC7A11*, thereby promoting cellular ferroptosis and exacerbating hepatic IRI, through the integration of multiomics sequencing. By thoroughly exploring the intricate regulatory relationships among YY1, NEDD4L, and SLC7A11, we aim to provide important insights for a comprehensive understanding of the pathogenic mechanisms of IRI, improve the effectiveness of IRI treatment, and develop more efficient therapeutic strategies. The significance of this research lies in providing more effective treatment methods for patients and driving advancements in clinical medicine. 

## Materials and Methods

### Establishment and management of a mouse hepatic IRI model

The experimental procedures and animal handling protocol were approved by the Institutional Animal Care and Use Committee of the Affiliated Huai’an No.1 People’s Hospital of Nanjing Medical University. The animal experiments in this study were conducted in accordance with internationally recognized animal welfare standards and relevant regulations. All possible measures were taken during the experiment to minimize animal suffering and discomfort. At the end of the experiment, the animals were anesthetized with isoflurane and then placed in a euthanasia chamber for CO _2_ euthanasia. 

Forty-six male C57BL/6J mice (aged 6–8 weeks and weighing 20 ± 2 g) were purchased from Beijing Vital River Laboratory Animal Technology Co., Ltd (No. 219; Beijing, China). The mice were housed in standard cages, maintained at a constant temperature of 23 ± 1°C with a 12-12 h light-dark cycle, and provided with food and water ad libitum. The mice underwent a one-week adaptation period before the experiment.

For the liver ischemia-reperfusion (I/R) model, the mice were fasted for 8–12 h before surgery. Anesthesia was induced via isoflurane, followed by laparotomy to expose the liver. Nontraumatic clamps were used to occlude the artery and portal vein of the left liver lobe, resulting in a 70% reduction in liver blood supply. After 90 min of ischemia, the clamps were released to allow for liver reperfusion. The relevant samples were harvested for analysis 24 h after reperfusion. In the sham-operated (sham) group, the mice underwent laparotomy and vascular dissection without I/R treatment. Postoperatively, the mice were placed on a temperature-controlled pad which was set at 37°C to maintain body temperature.

The mice were randomly divided into four groups, with 10 mice in each group. The experimental groups were oe-YY1-NC + sham, oe-YY1 + sham, oe-YY1-NC + I/R, and oe-YY1 + I/R. oe-YY1-NC and oe-YY1 were delivered via lentivirus infection, with virus packaging services provided by Sangon Biotech (Shanghai, China). Lentivirus treatment was administered two weeks before I/R model establishment through intrahepatic injection (1 × 10 ^1^ GC/mL, dissolved in 200 μL of saline solution, injected at 4 sites) [19]. 

### Liver tissue proteomics sequencing

Three Sham and three I/R mice were used for liver tissue proteomic sequencing. Mouse liver tissue proteins were extracted via RIPA buffer containing a protease inhibitor (P0013D; Beyotime, Shanghai, China). During extraction, the samples were sonicated for 30 s every 5 min for a total of three cycles to ensure cell lysis and protein release. The concentration of the extracted protein samples was determined via a BCA protein quantification kit (P0010; Beyotime), ensuring that each sample fell within an acceptable range according to the standard curve. Following pH adjustment to 8.0, trypsin was added at an enzyme-to-protein ratio of 1:50, and the mixture was incubated at 37°C for 16 h for enzymatic digestion. After digestion, the samples were cleaned via ZipTip C18 microcolumns (ZTC18S960; Millipore, Billerica, USA) and loaded onto a high-performance liquid chromatography system connected to a mass spectrometer for MS/MS analysis. Data processing, including protein identification and quantification, was performed via MaxQuant software.

The samples were labeled with iTRAQ for mass spectrometry analysis. Each sample was analyzed three times via a QSTAR Elite Hybrid MS (Applied Biosystems/MDS-SCIEX, Foster City, USA) and an online HPLC system (Shimadzu, Kyoto, Japan). During each analysis, 30 μL of peptide mixture was injected, separated on a custom nano-C18 column, and subjected to microliquid chromatography-electrospray ionization (75 μm ID × 15 cm, 5 μm particles) (New Objectives, Wubrun, USA). A 90-min HPLC gradient was established using mobile phase A (0.1% FA/2% ACN) and mobile phase B (0.1% FA/100% ACN) at an effective flow rate of 0.2 μL/min, with a constant flow of 30 μL/min at the column outlet. The mass spectrometer was operated in positive ion mode for data acquisition. Precursors with charges ranging from +2 to +4 were selected for fragmentation within the mass range of 300–2000 m/z. The three most abundant peptide ions with counts exceeding 5 were selected from each MS/MS spectrum. Dynamic exclusion was set for 30 s, with a mass tolerance of 30 mDa. The collision energy and automatic mass spectrometry/mass spectrometry accumulation activation were set automatically on the basis of ion abundance. The fragmentation intensity multiplier was set at 20, with a maximum accumulation time of 2 s. Three LC-MS/MS injections were performed (technical replicates = 3) to achieve better coverage of the target proteome and improved statistical consistency. Data validation of the proteomics results after iTRAQ labeling was conducted via western blot analysis of the same sample. The parameters used were as follows: (1) MS: scan range (m/z) = 350 –1500; resolution = 120000; AGC target = 4 × 10 ^5^; maximum injection time = 50 ms; (2) HCD-MS/MS: resolution = 30000; AGC target = 1 × 10 ^5^; collision energy = 33; and (3) DIA (data-independent acquisition), with each window overlapping by 1 m/z and a total of 47 windows. An iRT calibration kit (Ki3002; Biognosys AG, Zurich, Switzerland) was added for retention time calibration of the extracted peptide peaks. The DIA dataset was generated via Spectronaut V 13 (Biognosys AG), including data normalization and relative protein quantification [20]. 

### Proteomic data analysis and screening of IRI-associated proteins

The differentially expressed proteins (DEPs) between groups were screened via Welch’s ANOVA with the criteria of |log2FC| > 2 and *P* < 0.05 [21]. 

### High-throughput transcriptome sample sequencing, data quality control, and differential gene analysis

Total RNA from each sample was extracted via TRIzol reagent (16096020; Thermo Fisher Scientific, Waltham, USA). The RNA concentration, purity, and integrity were assessed via a Qubit® 2.0 Fluorometer® (Q33216; Life Technologies Gaithersburg, USA) with a Qubit® RNA analysis kit (HKR2106-01; BGI Biotechnology, Shanghai, China), a Nanodrop spectrophotometer (IMPLEN, Westlake Village, USA), and a Bioanalyzer 2100 system with an RNA Nano 6000 analysis kit (5067-1511; Agilent, Shanghai, China). The total RNA content for each sample was 3 μg, which served as the input material for RNA sample preparation. Following the manufacturer’s protocol, cDNA libraries were prepared via the NEBNext® UltraTM RNA library preparation kit for Illumina® (E7435L; NEB, Beijing, China) and evaluated for quality on a Bioanalyzer 2100 system (Agilent). The indexed samples were subsequently clustered on the cBot cluster generation system via the TruSeq PE Cluster Kit v3 cBot HS (PE-401-3001; Illumina, San Diego, USA) as recommended. After cluster generation, the library was sequenced on the HiSeq 550 platform (Illumina) to generate 125 bp/150 bp paired-end reads.

The quality of the paired-end reads of the raw sequencing data was examined via FastQC software v0.11.8 (Babraham Bioinformatics, Cambridge, UK). Preprocessing of the raw data was performed via Cutadapt software 1.18 to remove Illumina sequencing adapters and poly(A) tail sequences, and reads with more than 5% N content were filtered out via Perl script. Reads with a base quality of 20 or more covering 70% of the bases were extracted via FASTX Toolkit software 0.0.13, and then the paired-end sequences were further processed via BBMap software for repair. Finally, the filtered high-quality reads were aligned to the mouse reference genome via HISAT2 software (0.7.12) [ 22– 25]. 

### Cell culture and transfection

The AML12 normal mouse liver cell line (CRL2254; ATCC, Manassas, USA) was cultured in DMEM/F-12 (11320033; Thermo Fisher Scientific) supplemented with 10 μg/mL insulin, 5.5 μg/mL transferrin, 5 ng/mL selenium, 40 ng/mL dexamethasone, 10% FBS (10100147C; Thermo Fisher Scientific), and 1% penicillin-streptomycin (15140163; Thermo Fisher Scientific). The cells were maintained at 37°C in a humidified 5% CO _2_ incubator (51023126; Thermo Fisher Scientific). 

Cell lines with overexpression and knockdown of a specific gene were constructed via lentivirus-mediated methods along with their controls. The plasmids and lentivirus packaging services used were provided by Gene Engineering (Shanghai, China). In brief, plasmids carrying the target gene sequence/shRNA sequence were cotransfected with an auxiliary plasmid into 293T cells (CRL-3216; ATCC). After validation, amplification, and purification, packaged lentivirus was obtained. For lentivirus-mediated cell transfection, cells were seeded at a density of 5 × 10 ^5^ cells/well in 6-well plates. When the cell confluency reached 50%–70%, the cells were transfected with the appropriate amount of packaged lentivirus (MOI = 10, working titer of approximately 5 × 10 ^6^ TU/mL) and polybrene (5 μg/mL, TR-1003; Merck, Darmstadt, Germany). After 4 h, an equal amount of medium was added to dilute the polybrene. The medium was replaced by fresh medium after 24 h, and the cells were transfected via a fluorescent reporter gene after 48 h. Puromycin (1 μg/mL, A1113803; Thermo Fisher Scientific) was subsequently used for resistance selection to obtain stably transfected cell lines [26]. 

For knockdown cell line construction, two shRNA sequences were used, and the more efficient sequence was selected for subsequent experiments. The shRNA sequences are listed in Supplementary Table S1. For the cell groupings, sh-NEDD4L-NC refers to cells transfected with negative control short hairpin RNA corresponding to sh-NEDD4L, whereas sh-NEDD4L refers to cells transfected with specific short hairpin RNA targeting *NEDD4L* for the knockdown experiments. 

Cells treated with the ferroptosis inducer erastin (HY-15763; MCE, Monmouth Junction, USA) at a concentration of 10 μM were subjected to follow-up experiments after 24 h of treatment [27]. In this study, plasmids carrying Flag-tagged YY1 and its mutants (K339R and K341R) were obtained from Genechem Technology (Shanghai, China), while plasmids carrying HA-tagged Ub and its mutants (K48R and K63R) were obtained from HANYIN Bio-Technology (Shanghai, China). 

### Oxygen-glucose deprivation/reoxygenation (OGD/R)

AML12 cells were cultured in DMEM-F12 medium devoid of serum and glucose and then placed in an incubator at 37°C with 5% CO _2_ and 95% N _2_ for 12 h. After oxygen-glucose deprivation, the cells were returned to standard culture conditions to mimic *in vivo* reperfusion conditions. The cell samples were collected 24 h postreoxygenation for subsequent experiments unless otherwise specified [28]. 

For the experiments involving drug treatments, AML12 cells were treated with MG-132 (10 μM; MCE) for 4 h to inhibit proteasome activity and with leupeptin (50 μM; MCE) for 4 h to block protein degradation via the lysosomal pathway [29]. Each experiment was independently repeated three times. 

### Flow cytometry

Flow cytometric apoptosis analysis was performed via the Pacific Blue™ Annexin V/SYTOX™ AADvanced™ Apoptosis Detection Kit (A35136; Thermo Fisher Scientific) following the manufacturer’s instructions [28]. 

### Immuno-coimmunoprecipitation

Immunoimmunoprecipitation and ubiquitination analyses were performed via an immunoprecipitation kit (P2195M; Beyotime) following the manufacturer’s protocol. In brief, total lysates from whole tissues or cell extracts were collected and incubated with Protein G agarose gel bound with target protein antibodies or their controls (IgG) to create a gel-antibody suspension. The proteins were subsequently mixed at a ratio of 20 μL of gel-antibody suspension per 100 μL of protein sample and incubated overnight at 4°C on a shaker. After incubation, the gel was separated via centrifugation at 6000 *g* for 30 s at 4°C and washed, and the proteins were eluted via SDS-PAGE loading buffer for further analysis via western blot analysis [29]. The antibodies used in this experiment included anti-YY1 Ab (rabbit anti-mouse, 5 μg/mg of lysate) (ab245365; Abcam, Cambridge, UK), anti-NEDD4L Ab (rabbit anti-mouse, 5 μg/mg of lysate) (ab240753; Abcam), IgG (rabbit, 5 μg/mg of lysate) (ab172730; Abcam), and anti-Flag (rabbit, 1:30) (ab205606; Abcam). 

### Quantification of intracellular Fe ^2+^ content 

Cells were seeded in 12-well plates at a density of 1 × 10 ^5^ cells/mL. The levels of intracellular Fe ^2+^ ions were determined via an Iron Assay Kit (MAK025; Merck) following the manufacturer’s instructions. In brief, the cells were stained with 1 μM FerroOrange in HBSS at 37°C for 30 min. The fluorescence absorbance of the culture was subsequently measured immediately via a microplate reader (Synergy H1; BioTek Instruments, Winooski, USA) at an excitation wavelength of 543 nm and an emission wavelength of 580 nm [29]. 

### Detection of ferroptosis biomarkers

In accordance with the manufacturer’s protocol, the cells were collected and centrifuged in centrifuge tubes, after which the supernatant was discarded. One milliliter of extraction solution was added per 5 million cells, and the cells were sonicated at a power of 200 W for 3 s at 10 s intervals; this process was repeated 30 times. The supernatant was collected after centrifugation at 8000 *g* for 10 min at 4°C and placed on ice for analysis. For tissue sample preparation, approximately 0.1 g of tissue was weighed and homogenized in 1 mL of extraction solution on ice. After centrifugation at 8000 *g* for 10 min at 4°C, the supernatant was collected and placed on ice for analysis. A Micro Malondialdehyde (MDA) Assay Kit (BC0025; Solarbio, Beijing, China) was used to measure the MDA content in cells or tissues; a Lipid Peroxidation (4-HNE) Assay Kit (ab238538; Abcam) was used to detect the 4-HNE content in cells or tissues; and a GSH Assay Kit (Colorimetric) (ab239727; Abcam) was used to measure the GSH content [30]. The measurements were taken using a microplate reader (Varioskan LUX; Thermo Fisher Scientific) at the appropriate wavelengths. Specifically, readings of MDA were taken at 532 nm and 600 nm, those of 4-HNE were taken at 450 nm and 620 nm, and those of GSH were taken at 450 nm. 

### Transmission electron microscopy

The liver tissue was fixed in 1.25% glutaraldehyde/0.1 M phosphate buffer and postfixed in 1% OsO _4_/0.1 M phosphate buffer. After the samples were sectioned into 50-nm ultrathin sections via a microtome, they were placed on copper grids, stained with uranyl acetate and lead citrate, and examined under a transmission electron microscope (HT7800; HITACHI, Tokyo, Japan). To quantify mitochondrial damage, five random fields were evaluated for each sample [31]. 

### ELISA

The concentrations of ALT (ab282882), AST (ab263882), IL-1β (ab100704), TNF-α (ab208348), and MCP1 (ab208979) in mouse serum were quantified via ELISA using the corresponding kits from Abcam following the manufacturer’s protocols [32] .

### H&E staining

After deparaffinization of the liver tissue, the samples were rinsed in 1× PBS for 2 s, followed by hematoxylin staining (60°C, 60 s), another 10-s wash in 1× PBS, treatment with 1% hydrochloric acid alcohol differentiation solution for 3 s, and a subsequent 2-s wash in 1× PBS. Eosin staining was subsequently conducted for 3 min, followed by another 2-s wash in 1 × PBS. The samples were sequentially placed in 70%, 80%, and 95% ethanol, followed by a final dehydration step in absolute ethanol for 5 min. The samples were subsequently treated three times (5 min each) with xylene. Finally, the slides were mounted with mounting medium, and observations were made and images were captured via an optical microscope (CX43; Olympus, Tokyo, Japan) for further analysis and preservation [33]. 

### ChIP, DNA pulldown, and dual-luciferase reporter assays

For the cells that reached 70%–80% confluence, 1% formaldehyde was added to fix the cells at room temperature for 10 min to cross-link the DNA and proteins. Subsequently, sonication was performed to randomly disrupt the protein-DNA complexes, with 10 s on and 10 s off cycle for a total of 15 cycles to break them into appropriately sized fragments. The sample was then centrifuged at 12,000 *g* at 4°C, and the supernatant was divided into two parts, each incubated at 4°C overnight with negative control antibody IgG (1 μg/mL) (ab171870; Abcam) or target-specific antibody anti-YY1 Ab (rabbit anti-mouse, 2.5 μg/10 ^6^ cells) (MA5-32052; Thermo Fisher Scientific). The intrinsic DNA-protein complexes were precipitated via protein agarose/Sepharose, followed by removal of the supernatant after centrifugation, washing of nonspecific complexes, overnight decrosslinking at 65°C, and final extraction and purification of DNA fragments via phenol/chloroform. The products from ChIP-PCR were subjected to 3% agarose gel electrophoresis for qualitative analysis, and the primer sequences are detailed in Supplementary Table S2 .

AML12 cells were transfected with 50 nM biotin-labeled Biotin-SLC7A11-Wt (5′-CAAGAGGG-3′)/Biotin-SLC7A11-Mut (5′-GTTCTCCC-3′) (Jinkairui, Wuhan, China). After 48 h, the cells were collected, washed with PBS, and then lysed. The lysate was mixed with M-280 streptavidin magnetic beads precoated with BSA and yeast tRNA (55714; Merck, Rahway, USA) without RNase and incubated at 4°C for 3 h. Washes were performed twice with prechilled lysis buffer, three times with low-salt buffer, and once with high-salt buffer. Finally, the enrichment of the relevant proteins was assessed via western blot analysis [34]. 

Binding sites for the YY1 and *SLC7A11* promoter regions were predicted via the JASPAR database, and wild-type (Wt, 5′-CAAGAGGG-3′) and mutant (Mut, 5′-GTTCTCCC-3′) sequences of *SLC7A11* were constructed. These sequences were inserted into the pGL-3 luciferase reporter vector (4351372; Thermo Fisher Scientific) and transfected into oe-YY1-NC or oe-YY1 cells constructed from AML12 cells. After 48 h, the cells were collected, lysed, and centrifuged at 250 *g* for 3‒5 min, and the supernatant was used for the measurement of luciferase activities using the Dual-Luciferase Reporter Assay System (E1910; Promega, Madison, USA). A total of 100 μL of firefly luciferase working solution was added to each cell sample for firefly luciferase measurement, and another 100 μL of Renilla luciferase working solution was added for Renilla luciferase measurement. The ratio of firefly to Renilla luciferase activity was used as the relative luciferase activity [35]. Each experiment was repeated three times. 

### Immunohistochemistry

The tissue samples were fixed in 4% paraformaldehyde, dehydrated in ethanol, cleared in xylene, and embedded in paraffin for sectioning. Prior to incubation with the primary antibodies, the sections were rehydrated and subjected to antigen retrieval according to the instructions provided by the antibody manufacturer. Immunostaining was performed via a universal two-step detection kit (PV-9000; Zhongshan Golden Bridge, Beijing, China) according to the manufacturer’s protocol. The staining results were observed and documented via an optical microscope (CX43; Olympus). A semiquantitative analysis of the results was conducted via Image-Pro Plus software [36]. The primary antibodies used in this experiment included an anti-Ly6G antibody (rabbit anti-mouse, 1:2000) (ab238132; Abcam) and an anti-NEDD4L antibody (rabbit anti-mouse, 1:100) (ab240753; Abcam). 

### Western blot analysis

Total protein was extracted from the samples via a protein extraction kit (BB3101; Bestbio, Shanghai, China), and the protein concentration was determined via a BCA assay kit (P0012S; Bestbio). A 10% SDS-PAGE gel (P0012A; Bestbio) was prepared for the experiments. Each well was loaded with 50 μg of protein sample, and electrophoresis was conducted at a constant voltage of 80 V to 120 V for 2 h, followed by a constant current of 250 mA for 90 min to transfer the proteins to a PVDF membrane (IPVH00010; Merck). The PVDF membrane was blocked with 5% skim milk in TBST at room temperature for 2 h and then washed with TBST for 10 min to remove excess reagents. The membrane was subsequently incubated overnight at 4°C with the appropriate primary antibody, followed by washing with TBST to remove excess primary antibody. Goat anti-rabbit IgG H&L (HRP) (1:2000, ab6721; Abcam) or goat anti-mouse IgG H&L (HRP) (1:2000, ab6789; Abcam) was then added and incubated at room temperature for 1 h, followed by washing to remove excess secondary antibody. Finally, the membrane was visualized via an enhanced chemiluminescence (ECL) reagent (P0018FS; Bestbio) and the results were quantitatively analyzed via Image-Pro Plus 6.0 software (Media Cybernetics, Silver Springs, USA). Each sample was tested in triplicate. GAPDH was used as an internal control. The details of the antibodies used are shown in Supplementary Table S3. 

### RT-qPCR

Total RNA was extracted from the samples via TRIzol (16096020; Thermo Fisher Scientific), after which the reaction system was set up with the One Step TB Green® PrimeScript™ RT-PCR Kit (RR066A; Takara, Shiga, Japan) for one-step reverse transcription and PCR. RT-qPCR was run on a Thermal Cycler Dice™ Real-Time System III (TP990; Takara) with a program consisting of a reverse transcription stage (42°C for 5 min, 95°C for 10 s, cycle number = 1), a PCR stage (95°C for 5 s, 60°C for 34 s, cycle number = 40), and a melting curve stage (95°C for 15 s, 60°C for 1 min, 95°C for 15 s, cycle number = 1). The amplification and melting curves were validated after the reactions. The 2 ^‒ΔΔCt^ method was employed to calculate the relative ratio of target gene expression between the experimental and control groups, where ΔΔCT = ΔCt _experimental_ ‒ ΔCt _control_, and ΔCt = Ct _target_ ‒ Ct _reference_. Ct represents the amplification cycle number required to reach the set threshold of real-time fluorescence intensity [37]. Each sample was subjected to 3 replicate wells in each experiment, and the experiments were repeated 3 times. The primer sequences are listed in Supplementary Table S4, and *GAPDH* was used as the reference gene. 

### Statistical analysis

Bioinformatics results were statistically analyzed via R 4.3.0, whereas other data were analyzed via SPSS 26.0 (IBM, Armonk, USA). Continuous data are expressed as the mean ± standard deviation; for differential analysis of the data, normality and homogeneity of variance tests were conducted first. If the data followed a normal distribution and had homogenous variances, Student’s *t* test was used to calculate the differences. Otherwise, nonparametric tests were employed. A *P* value less than 0.05 was considered statistically significant. 

## Results

### Downregulation of YY1 protein expression in IRI indicates its role in injury progression

**Figure 1:**
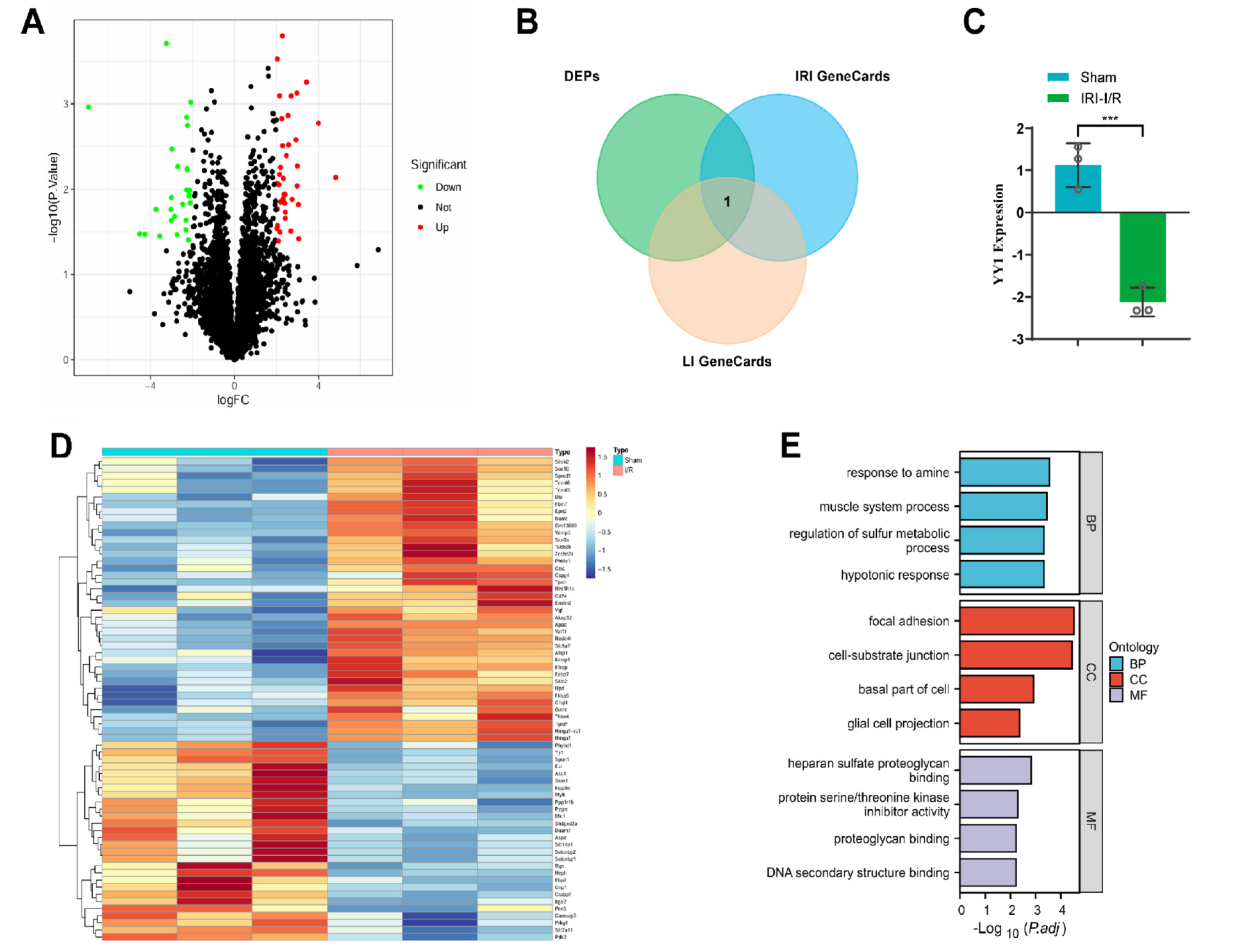
Figure 1 Downregulation of the YY1 protein in IRI and analysis of its potential role (A) Volcano plot depicting differential protein expression. (B) Intersection of gene sets. (C) Expression of YY1 in the proteomic analysis. n = 3 in each group. ***P < 0.001. (D) Heat map of differential proteomic analysis results. n = 3 per group. (E) Enrichment analysis results of DEGs in GO categories. BP: biological process; CC: cellular component; MF: molecular function.

To investigate key factors involved in hepatic IRI, we established a mouse model of liver I/R. Liver tissue samples from the IRI-I/R group were harvested 24 h postreperfusion, with liver tissue samples from the sham group serving as controls for proteomic sequencing. After data quality control and filtering, DEPs were selected on the basis of the criteria |log2FC| > 2 and *P* < 0.05. The results revealed 68 DEPs between the sham and IRI-I/R groups, 40 of which were upregulated and 28 of which were downregulated in the IRI-I/R group ( Figure 1A,D). Functional enrichment analysis revealed that these 68 DEPs were enriched mainly in GO terms such as response to amine, muscle system process, and hypotonic response ( Figure 1E). We subsequently retrieved 2062 and 9696 relevant genes from the GeneCards website via the keywords “ischemia-reperfusion injury” and “liver injury”, respectively, and selected the top 2000 scored genes for further analysis (referred to as IRI GeneCards and LI GeneCards). By intersecting the gene sets obtained from GeneCards with the DEPs (to align the research more closely with clinical applications, we converted the mouse-derived DEPs to their corresponding human gene names), we identified one gene, *YY1*, closely related to IRI ( Figure 1B), which was downregulated in IRI-I/R ( Figure 1C). These results indicate a significant decrease in YY1 protein expression in IRI liver tissue, suggesting its involvement in the progression of IRI.

### YY1 overexpression alleviates IRI *in vitro* and *in vivo*

**Figure 2:**
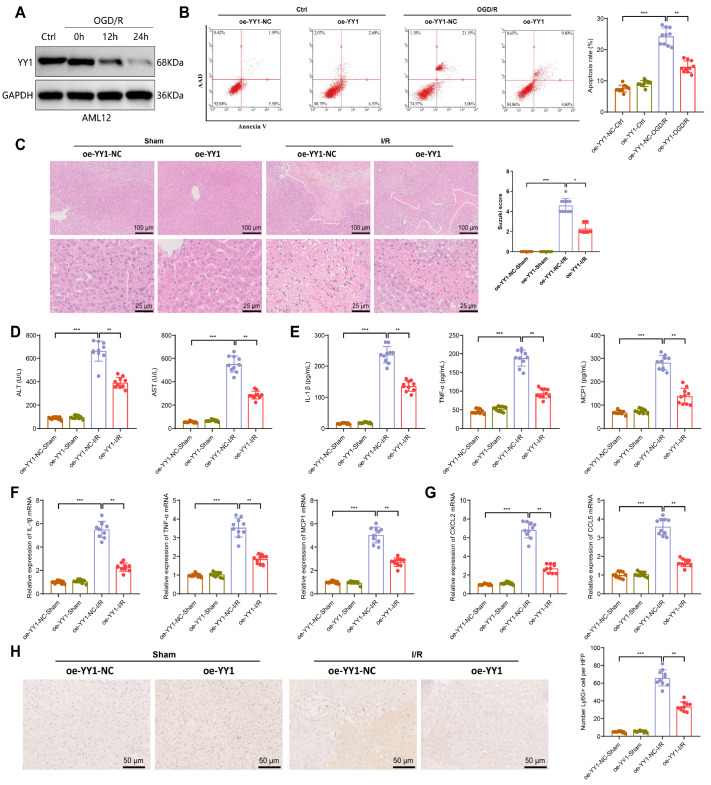
Figure 2 Impact of YY1 overexpression on *in vitro* and *in vivo* hepatic IRI (A) Western blot analysis of AML12 YY1 expression post IRI-OGD/R treatment. (B) Flow cytometry assessment of the effect of YY1 overexpression on IRI-OGD/R-induced apoptosis, with representative results on the left and corresponding bar graph on the right. (C) Evaluation of liver tissue damage in Sham and IRI-I/R treated mice following YY1 overexpression, with representative H&E staining results on the left showing a low magnification view, white dashed lines indicating the injury area (scale bar = 100 μm), and local enlargement of the injury area (scale bar = 25 μm), and Suzuki scores on the right. (D) ELISA results of serum ALT and AST levels in each group of mice. (E,F) Expression levels of the inflammatory factors IL-1β, TNF-α, and MCP1 in mouse serum and liver tissue within each group assessed by ELISA and RT-qPCR. (G) Expression levels of the chemokines CXCL2 and CCL5 in mouse liver tissues in each group measured by RT-qPCR. (H) Immunohistochemistry was performed to evaluate the infiltration of Ly6G+ neutrophils in the liver tissues of different groups of mice. A representative image is presented on the left, accompanied by a corresponding statistical bar graph on the right (scale bar = 50 μm). n = 10 in each group for the animal experiments. Cell experiments were repeated three times. *P < 0.05, **P < 0.01, ***P < 0.001. ns, indicates no significance.

After confirming the variation in YY1 expression in hepatic IRI, we investigated this relationship. We used a mouse liver cell line, AML12, subjected to oxygen and glucose deprivation/reperfusion (OGD/R) to simulate *in vivo* IRI (referred to as IRI-OGD/R) and then detected the expression of YY1 at 0 h, 12 h, and 24 h postreperfusion. Consistent with the results of the animal experiments, YY1 expression in AML12 cells was significantly downregulated at the protein level after IRI-OGD/R treatment, with the most significant downregulation observed at 24 h postreoxygenation ( Figure 2A), although no significant change was observed at the transcriptional level ( Supplementary Figure S1A). We subsequently examined the impact of *YY1* overexpression or knockdown ( Supplementary Figure S1B,C) on IRI-OGD/R-induced cell apoptosis. Flow cytometry results demonstrated that YY1 overexpression significantly inhibited IRI-ODG/R-induced cell apoptosis ( Figure 2B), whereas *YY1* knockdown notably promoted cell apoptosis induced by IRI-OGD/R ( Supplementary Figure S1 D).

Furthermore, we validated our findings through *in vivo* experiments. By upregulating YY1 expression in liver tissues via lentivirus (LV) treatment ( Supplementary Figure S1E), we established an IRI-I/R model. The results revealed that YY1 overexpression significantly alleviated liver damage induced by IRI-I/R. Specifically, compared with the oe-YY1-NC group, the oe-YY1 group presented more regular liver tissue morphology; significantly lower Suzuki scores ( Figure 2C); lower serum ALT and AST levels ( Figure 2D); and lower levels of the serum and tissue inflammatory factors IL-1β, TNF-α, and MCP1 ( Figure 2E,F). The recruitment and activation of neutrophils play crucial roles in hepatic IRI [38]. Assessment of hepatic chemokine expression and neutrophil infiltration revealed that, compared with the oe-YY1-NC group, the oe-YY1 group displayed significantly reduced expression of chemokines ( Figure 2G) and decreased Ly6G ^+^ neutrophil counts ( Figure 2H). In summary, the above results suggest that overexpression of YY1 significantly alleviates liver cell and liver tissue IRI both *in vitro* and *in vivo* .

### Upregulation of NEDD4L expression by IRI-OGD/R induces YY1 ubiquitination and degradation

**Figure 3:**
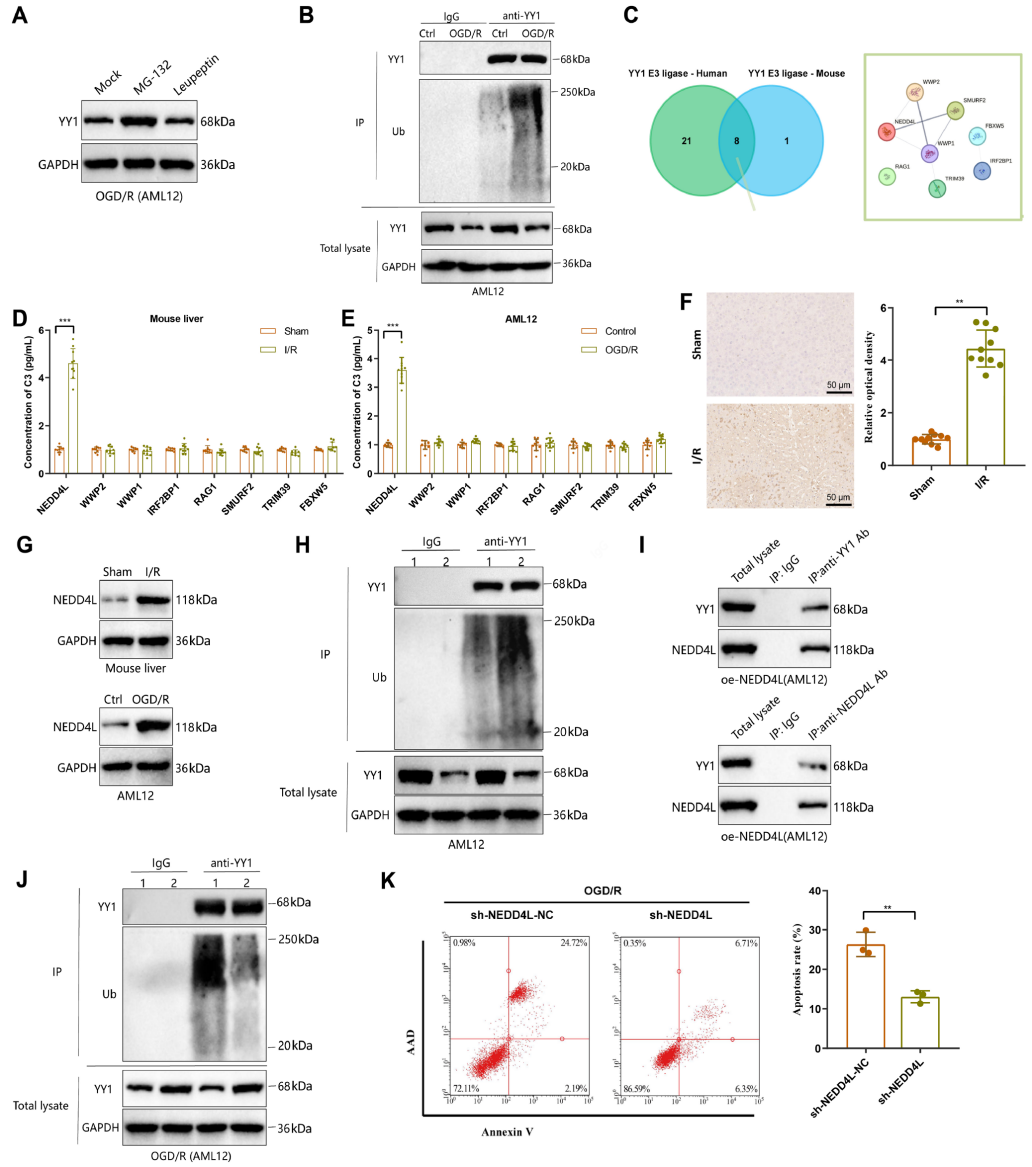
Figure 3 Regulatory effect of NEDD4L expression in IRI on the ubiquitination-induced degradation of YY1 (A) Influence of MG-132 and leupeptin treatments on the reduction in YY1 expression induced by IRI-OGD/R, as detected by western blot analysis. (B) Detection of YY1 ubiquitination levels after IRI-OGD/R treatment. (C) Intersection of predicted human and mouse upstream E3 ligases of YY1, Venn diagram on the left, and the PPI network of 8 intersecting E3 ligases on the right (interaction score = 0.15). (D,E) Expression levels of 8 E3 ligases in mouse liver tissues and the AML12 cell line after IRI detected by RT-qPCR. (F) Immunohistochemical analysis of NEDD4L expression in mouse liver tissues after IRI-I/R treatment, representative images on the left, and quantitative statistical results on the right (scale bar = 50 μm). (G) Expressions of NEDD4L in mouse liver tissues and the AML12 cell line after IRI detected by western blot analysis. (H) Detection of YY1 expression and ubiquitination levels in AML12 cells overexpressing NEDD4L. (I) Co-IP experiment was used to detect the interaction between YY1 and NEDD4L in oe-NEDD4L. (J) Western blot analysis of the effects of NEDD4L knockdown on YY1 ubiquitination and expression after IRI-OGD/R treatment. (K) Flow cytometry analysis of the effects of NEDD4L knockdown on cell apoptosis induced by IRI-OGD/R; n = 10 per group in animal experiments. Cell experiments were repeated three times. *P < 0.05, **P < 0.01, ***P < 0.001.

During the validation process, we observed a significant decrease in YY1 protein expression following IRI (including I/R and OGD/R) treatment, whereas *YY1* mRNA expression remained unaffected, suggesting that IRI may specifically promote YY1 degradation at the protein level. To investigate the pathway through which IRI facilitates YY1 degradation, we treated AML12 cells with MG-132 (a proteasome inhibitor) and leupeptin (a lysosomal pathway inhibitor). The results indicated that treatment with MG-132, but not leupeptin, reversed the reduction in YY1 protein expression induced by IRI-OGD/R ( Figure 3A), indicating the involvement of the proteasome pathway in IRI-OGD/R-induced YY1 degradation. Ubiquitination is a major pathway for proteasomal degradation [39]; therefore, we examined the ubiquitination status of YY1 after IRI-OGD/R. Coimmunoprecipitation revealed a significant increase in the level of ubiquitin bound to YY1 in the IRI-OGD/R group compared with that in the control group, indicating that IRI-OGD/R indeed induced the ubiquitination of YY1 ( Figure 3B).

Ubiquitination is orchestrated through the E1-E2-E3 enzymatic cascade [40]. In contrast to the relatively conserved E1 and E3 enzymes, the diverse members of the E3 family exhibit specificity in recognizing different substrates, thus demonstrating high selectivity for protein degradation [41]. Therefore, following the confirmation of YY1 ubiquitination in the process of IRI-OGD/R, we explored the types of E3 ligases that regulate this process. The prediction of E3 ligases regulating YY1 ubiquitination was conducted via the UbiBrowser 2.0 database ( http://ubibrowser.bioit.cn/ubibrowser_v3/). The results revealed that 29 and 9 E3 ligases in humans and mice, respectively, are capable of modulating YY1 ubiquitination ( Supplementary Figure S2A). To align the research findings with clinical applications, a convergence of the predicted resuplts for humans and mice revealed 8 E3 ligases, namely, NEDD4L, WWP2, WWP1, IRF2BP1, RAG1, SMURF2, TRIM39, and FBXW5 ( Figure 3C). We validated the expressions of these 8 E3 ligases under IRI conditions. The results demonstrated that only NEDD4L was significantly upregulated post-IRI in both tissues and cells ( Figure 3D,E). Further protein validation revealed significant uregulation of NEDD4L expression in mouse liver tissue and AML12 cells following IRI (I/R or OGD/R) treatment ( Figure 3F,G), suggesting that the upregulation of NEDD4L in IRI may play a critical role in the ubiquitination and degradation of YY1. 

We subsequently established AML12 cells overexpressing NEDD4L ( Supplementary Figure S2B) and assessed the expression of YY1. The results indicated that NEDD4L overexpression significantly increased the level of ubiquitinated YY1, leading to decreased YY1 protein expression ( Figure 3H) without affecting *YY1* mRNA level ( Supplementary Figure S2C). Coimmunoprecipitation experiments demonstrated that in oe-NEDD4L cells, NEDD4L directly interacted with YY1 ( Figure 3I), suggesting that the ubiquitination of YY1 is mediated by NEDD4L. 

Furthermore, we knocked down *NEDD4L* in AML12 cells ( Supplementary Figure S2D) and subjected them to IRI-OGD/R treatment. Compared with sh-NEDD4L-NC, sh-NEDD4L reversed the IRI-OGD/R-induced ubiquitination and downregulation of YY1 expression ( Figure 3J) while also alleviating cell apoptosis ( Figure 3K). In summary, these results suggest that during IRI, the E3 ligase NEDD4L is upregulated and promotes the ubiquitination and degradation of YY1. 

### Upregulation of IRI and its associated NEDD4L promotes K63-linked ubiquitination of YY1 at position K339

After revealing that NEDD4L can regulate the ubiquitination of YY1, we investigated the predominant types of ubiquitination present in YY1. Previous studies have indicated that K48- and K63-linked polyubiquitin chains are the most abundant and functionally distinct forms of ubiquitination [29]. Therefore, we used arginine-substituted ubiquitin mutants at the K48 and K63 sites (HA-Ub-K48R and HA-Ub-K63R) to specifically block the formation of K48- and K63-linked ubiquitin chains. Cotransfection of wild-type (HA-Ub-WT) or mutant sequences with Flag-YY1 into AML12 cells followed by co-IP analysis revealed that when the FLAG-YY1 precipitation levels were consistent, the inhibitory effect of HA-Ub-K63R on the accumulation of ubiquitinated Flag-YY1 was most significant ( Supplementary Figure S3A), indicating that the predominant type of ubiquitinated YY1 involved K63-linked ubiquitin chains. 

**Figure 4:**
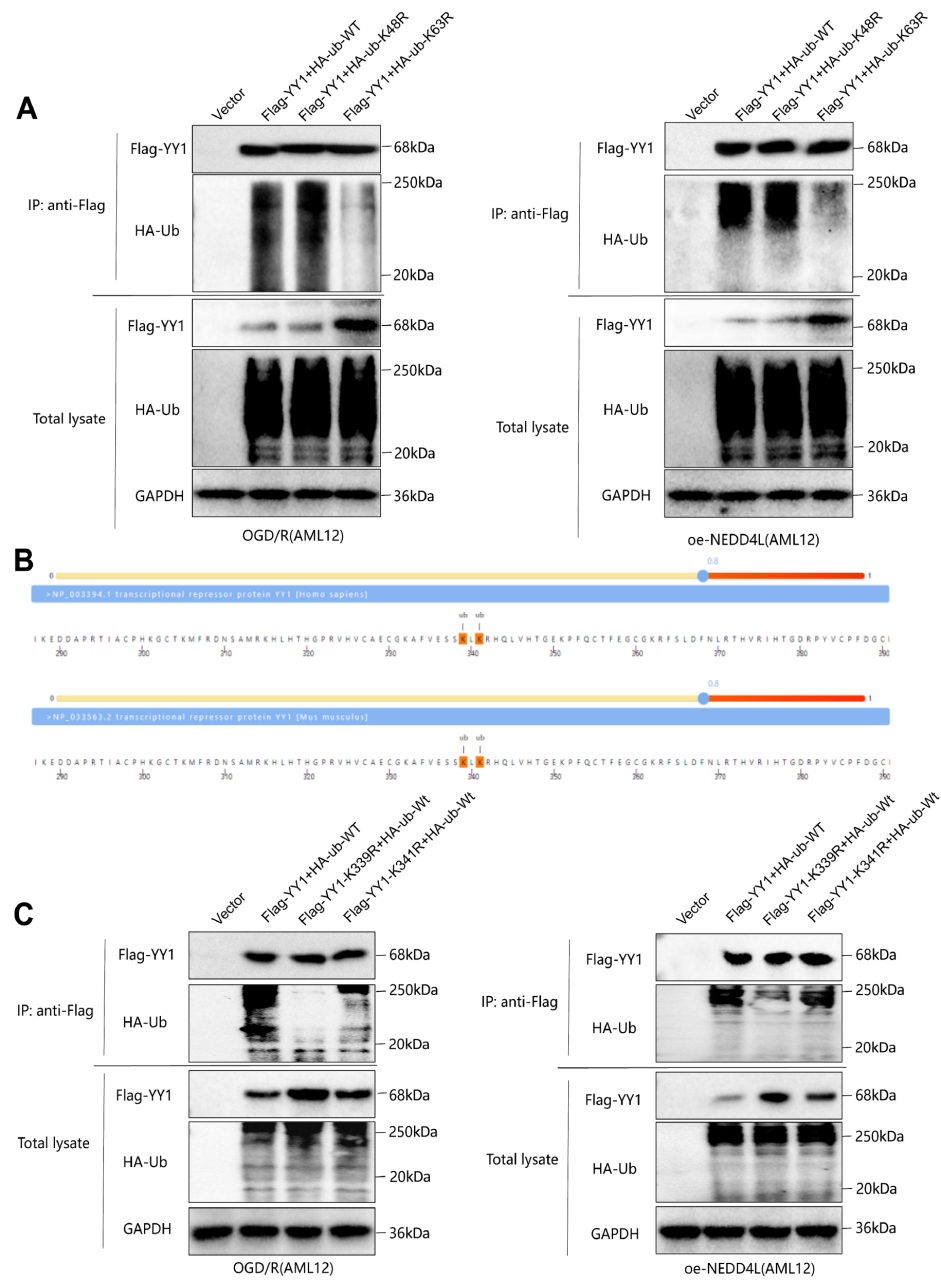
Figure 4 Validation of the major ubiquitination types and sites of YY1 in IRI (A) Detection of the impact of Ub K48 and K63 site mutations on YY1 ubiquitination expression levels under IRI-OGD/R (left) and NEDD4L overexpression (right) conditions by co-IP and western blot analysis. (B) Prediction of the highest-scoring K339 and K341 ubiquitination sites of human and mouse YY1 in MusiteDeep online tool results. (C) Detection of the influence of YY1 K339 and K341 site mutations on YY1 ubiquitination expression levels under IRI-OGD/R (left) and NEDD4L overexpression (right) conditions. Cell experiments were repeated three times.

We subsequently verified whether IRI and NEDD4L similarly promote the K63-linked ubiquitination of YY1. The results demonstrated that upon the induction of IRI-OGD/R or the overexpression of NEDD4L in AML cells, with consistent levels of Flag-YY1 precipitation, HA-Ub-K63R also had the most pronounced inhibitory effect on the accumulation of ubiquitinated Flag-YY1 ( Figure 4A), suggesting that the upregulation of IRI and its associated NEDD4L promoted the K63-linked ubiquitination of YY1.

Following the investigation of the main types of ubiquitination, we further explored the ubiquitination sites on YY1. The prediction of potential ubiquitination sites on YY1 via the MusiteDeep online tool revealed that both the human and mouse YY1 proteins have high-scoring ubiquitination sites at K339 and K341 (PTM scores = 0.823, the same scores for humans and mice at the same sites) ( Figure 4 B).

We subsequently mutated the K339 and K341 sites (K mutated to R) and cotransfected plasmids carrying the mutant sequences (Flag-YY1-K339R and Flag-YY1-K341R) with HA-Ub-WT into AML12 cells, with Flag-YY1-WT used as a control. Compared with transfection with Flag-YY1-WT, cotransfection with Flag-YY1-K339R or Flag-YY1-K341R led to a decrease in the ubiquitination level of Flag-YY1, with the most significant reduction observed in the Flag-YY1-K339R group ( Supplementary Figure S3B). Similarly, we validated that YY1 ubiquitination sites are regulated by IRI and NEDD4L. The results indicated that after the YY1 K339 site was mutated, the levels of Flag-YY1 ubiquitination induced by IRI and NEDD4L significantly decreased, and the degradation of Flag-YY1 was inhibited ( Figure 4C). Notably, although to a lesser extent than K339, the mutation of the YY1 K341 site also partially reversed the effects induced by the overexpression of IRI and NEDD4L, suggesting that other YY1 sites, including K341, are also subject to ubiquitination by IRI. Overall, these results demonstrate that the upregulation of IRI and its associated protein NEDD4L predominantly promotes the K63-linked ubiquitination of YY1 at the K339 site, thereby facilitating its degradation. 

### The NEDD4L/YY1 signaling axis regulates IRI-associated ferroptosis

**Figure 5:**
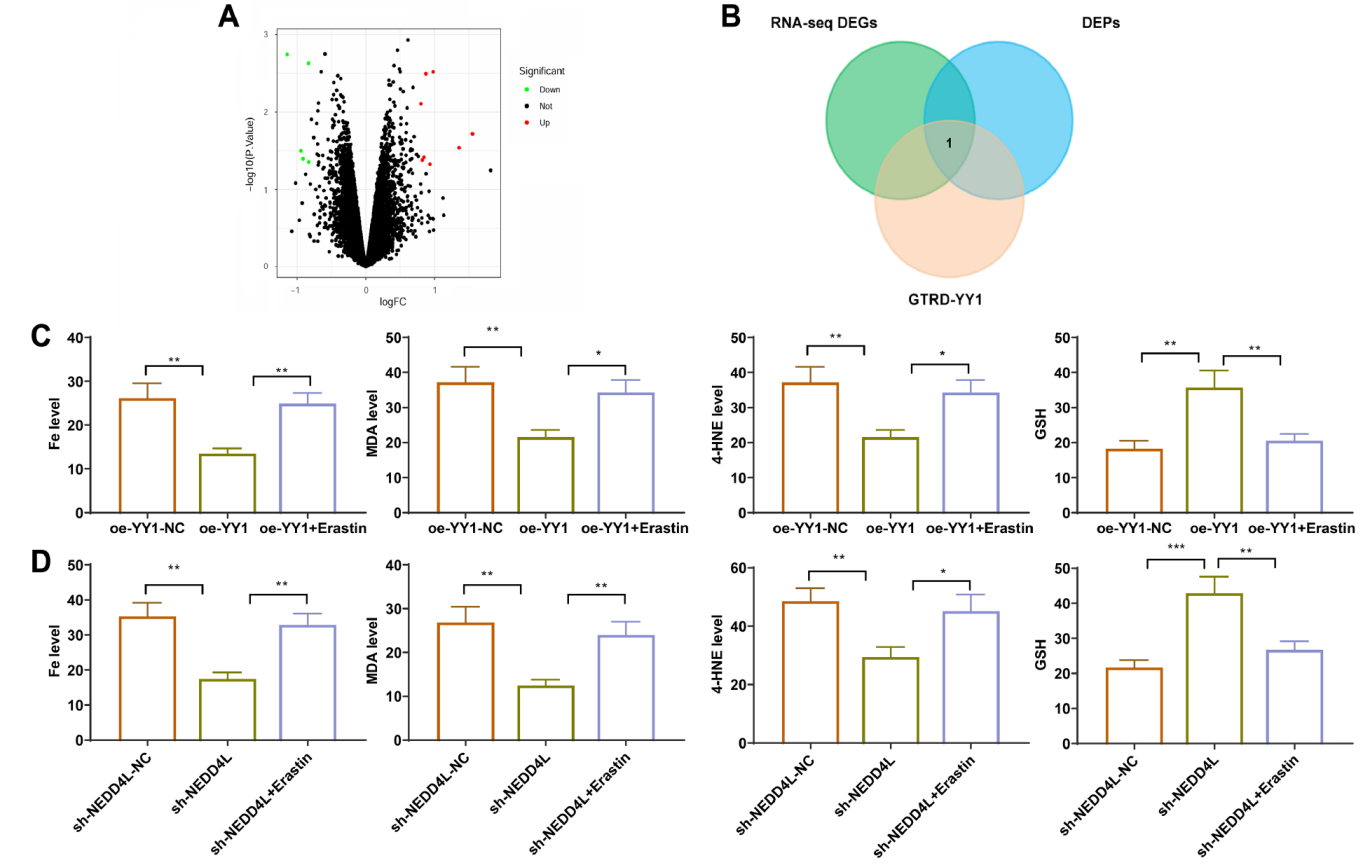
Figure 5 Validation of the regulatory role of the NEDD4L/YY1 signaling axis in IRI-related ferroptosis (A) Volcano plot of differentially expressed genes identified via oe-YY1 transcriptome sequencing analysis. (B) Intersection of gene sets. (C,D) Effects of YY1 overexpression, NEDD4L knockdown, and the use of the iron death inducer erastin on iron death induced by IRI-OGD/R. Cell experiments were repeated three times. *P < 0.05, **P < 0.01, ***P < 0.001. ns, indicates no significance.

To investigate the downstream target genes regulated by YY1 transcription and their potential mechanisms of action, we performed high-throughput transcriptome sequencing (RNA-seq) on AML12 cells overexpressing YY1 and their controls. After data quality control and filtering, we used the criteria of |log2FC| > 0.8 & *P* < 0.05 to select DEGs between the groups. The results revealed 23 DEGs (RNA-seq DEGs) between oe-YY1-NC and oe-YY1, with 17 upregulated and 6 downregulated in oe-YY1 ( Figure 5A). We subsequently intersected the RNA-seq DEGs with DEPs (both converted to human genes) and the predicted downstream target genes of YY1 from the GTRD database ( http://gtrd.biouml.org/#!) to ultimately identify one IRI-related downstream target gene of YY1, *SLC7A11* ( Figure 5B).

Studies have shown that SLC7A11 plays a crucial role in the regulation of ferroptosis [ 42, 43]. Ferroptosis plays a vital role in the progression of IRI [ 44, 45]. Therefore, we investigated whether YY1 and its regulation of expression through NEDD4L are involved in the progression of IRI through the regulation of ferroptosis. Upon overexpression of YY1 after IRI-OGD/R, we observed significant inhibition of ferroptotic cell death induced by IRI-OGD/R, as evidenced by notable decreases in the levels of intracellular iron, MDA and 4-HNE, and a significant increase in GSH expression. Conversely, treatment with the ferroptosis inducer erastin in combination led to significant increases in the levels of intracellular iron, MDA and 4-HNE, and a decrease in the GSH level ( Figure 5C). Similarly, knockdown of *NEDD4L* after IRI-OGD/R treatment yielded comparable outcomes ( Figure 5D). These findings indicate that the NEDD4L/YY1 signaling axis is involved in the regulation of IRI-associated ferroptosis. 

### YY1 positively regulates the transcription of the ferroptosis-related gene *SLC7A11*

**Figure 6:**
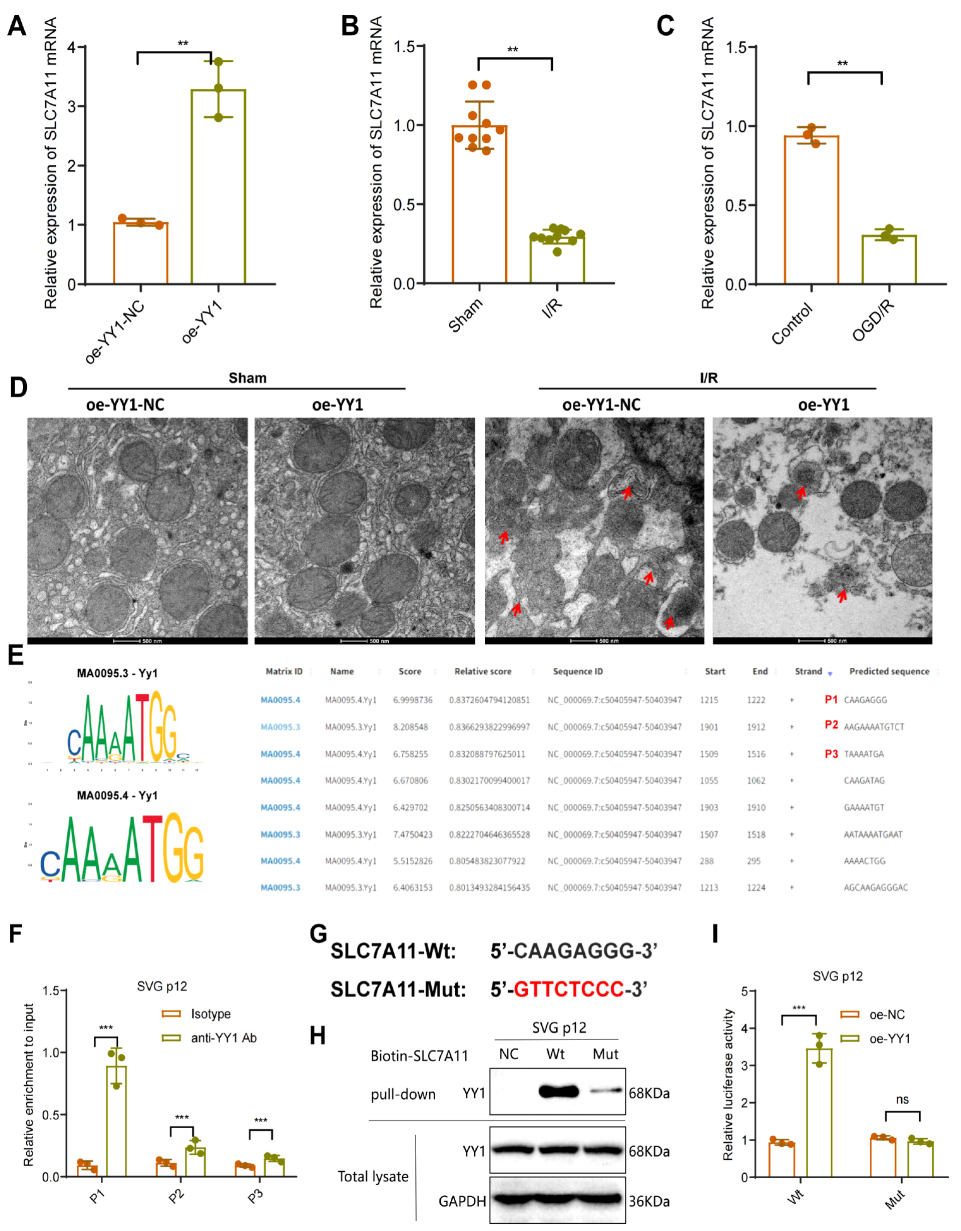
Figure 6 Validation of the transcriptional regulation of SLC7A11 by YY1 (A) Detection of SLC7A11 expression after YY1 overexpression via RT-qPCR. (B,C) Detection of SLC7A11 expression after IRI via RT-qPCR. (D) Transmission electron microscopy observation of mitochondrial morphology in mouse liver cells. (E) Prediction of binding sites between mouse YY1 and the SLC7A11 promoter region. (F) Validation of site binding via ChIP. (G) Wt and Mut sequences of site P1. (H,I) DNA pull-down and dual-luciferase reporter experiments verifying YY1’s transcriptional regulation of SLC7A11; animal experiments with n = 10 per group. Cell experiments were repeated three times. **P < 0.01, ***P <0.001. ns, indicates no significance.

We subsequently validated the regulatory role of YY1 in SLC7A11 expression. The results demonstrated significant upregulation of SLC7A11 expression in liver tissue cells upon overexpression of YY1 ( Figure 6A), suggesting its regulation by YY1. An evaluation of liver tissue from IRI model mice revealed markedly lower SLC7A11 expression in the IRI-I/R and IRI-OGD/R groups than in the sham and control groups ( Figure 6B,C), indicating the involvement of SLC7A11 in the IRI process. Additionally, transmission electron microscopy revealed that liver cell mitochondria in the oe-YY1-NC+ I/R group were smaller in size and had greater membrane density than those in the oe-YY1-NC + sham group; however, the overexpression of YY1 alleviated liver cell ferroptosis induced by IRI ( Figure 6D).

We further verified whether YY1 directly regulates the transcription of *SLC7A11*. The upstream promoter region of *SLC7A11* was obtained from NCBI (>NC_000069.7:c50405947-50403947 *Mus musculus* strain C57BL/6J chromosome 3, GRCm39), and via the JASPAR database ( https://jaspar.genereg.net/), with a relative profile score threshold of 80%, we predicted the binding of YY1 to this region. The results revealed the presence of 8 potential binding sites of YY1 within the upstream promoter region of *SLC7A11* ( Figure 6E). We validated the top three sites with high enrichment scores (designated P1--P3). ChIP experiments confirmed the binding of YY1 to all three sites, with the most significant enrichment observed at the P1 site ( Figure 6F), suggesting that the P1 site is the main binding site through which YY1 regulates the transcription of *SLC7A11*. We subsequently introduced mutations to the P1 sequence on the basis of the wild-type sequence (SLC7A11-Wt) to create the mutated sequence (SLC7A11-Mut) ( Figure 6G) and further validated the binding of YY1 to it. The results of the DNA pull-down experiments revealed a significantly lower level of YY1 binding to the SLC7A11-Mut sequence than to the SLC7A11-Wt sequence ( Figure 6H); furthermore, the luciferase reporter assay results demonstrated that YY1 overexpression significantly enhanced luciferase activity in the SLC7A11-Wt group but had no effect on the SLC7A11-Mut group ( Figure 6I), suggesting that YY1 in AML12 cells can regulate the transcription of *SLC7A11* through the P1 site. These findings indicate that YY1 positively regulates the transcription of SLC7A11, thereby inhibiting the occurrence of IRI-related ferroptosis. 

## Discussion

This study elucidates the molecular mechanism by which NEDD4L degrades YY1 to inhibit the transcription of *SLC7A11* through ubiquitination, which plays a crucial regulatory role in hepatic IRI. Initially, we validated the regulatory mechanism of NEDD4L on YY1 and reported that NEDD4L promotes the ubiquitination-mediated degradation of the YY1 protein, thereby suppressing the transcription of *SLC7A11*, consequently leading to the occurrence of ferroptosis and worsening the severity of liver injury. Concurrently, we compared the literature on the role of NEDD4L in other diseases or physiological processes and observed that this study is the first to describe the novel mechanism by which NEDD4L promotes ferroptosis by regulating YY1 in hepatic IRI [ 46– 48]. 

In our investigations, we noted a decrease in YY1 expression in liver tissues, which is primarily localized in liver cells. Overexpression of YY1 significantly alleviated hepatic IRI in liver tissues and cells, suggesting a protective role of YY1 in this context [ 49– 51]. By contrasting the literature regarding the role of YY1 in other tissues or diseases, we confirmed the unique regulatory function of YY1 in liver injury, laying a foundation for further exploration of its mechanism and clinical applications [ 49, 52]. 

Further studies revealed the significant impact of NEDD4L on liver ferroptosis. NEDD4L accelerates the proteasomal degradation of YY1 by promoting K63-linked ubiquitination at the K339 site, thereby inducing ferroptosis and exacerbating liver injury [ 47, 53, 54]. Compared with previous studies on the functions of E3 ligases in other contexts, we found that NEDD4L plays a distinctive regulatory role in hepatic IRI, providing crucial insights into the mechanism of ferroptosis [ 14, 55, 56]. 

The impact of YY1 on liver injury is further highlighted through the regulation of the downstream target gene *SLC7A11*. YY1 can alleviate the severity of liver injury by inhibiting ferroptosis induced by IRI [11]. After exploring the transcriptional regulatory role of YY1 in other pathways or pathological conditions, we confirmed the specificity of the role of YY1 in hepatic IRI, offering new insights into strategies for inhibiting ferroptosis [52] .

**Figure 7:**
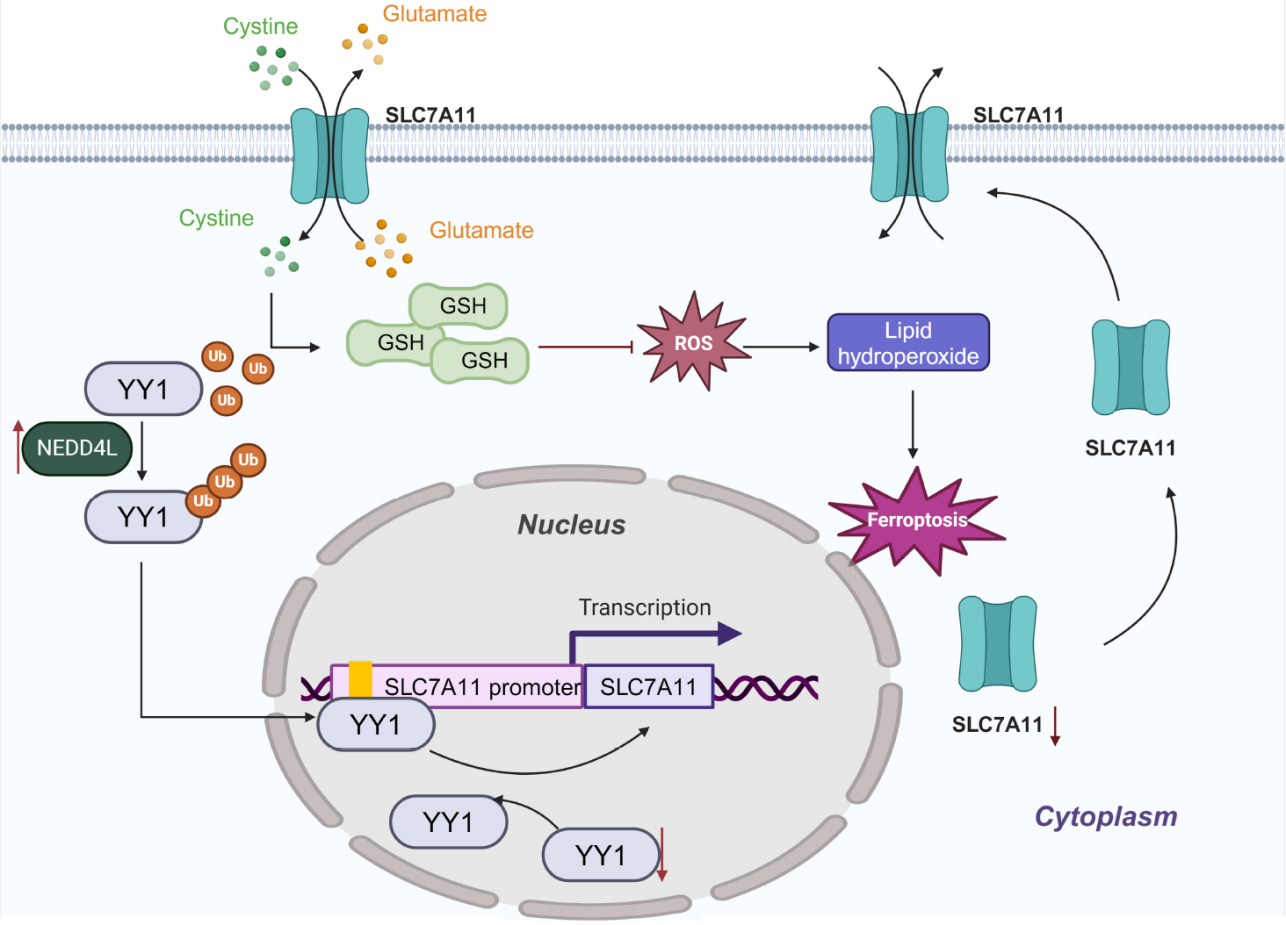
Figure 7 Molecular mechanism by which NEDD4L/YY1/SLC7A11 regulates IRI The E3 ubiquitin ligase NEDD4L promotes the ubiquitination and degradation of the YY1 protein, inhibiting the transcription of the ferroptosis inhibitor SLC7A11, thereby promoting the occurrence of IRI-related ferroptosis and exacerbating liver injury.

On the basis of the bioinformatics analysis and experimental validation conducted here, we preliminarily draw the following conclusions: YY1 is downregulated in liver tissues during hepatic IRI and is expressed mainly in liver cells. YY1 overexpression alleviated liver tissue and liver cell IRI both *in vitro* and *in vivo*. Further research revealed that during the IRI process, the expression of E3 ligases is upregulated, which promotes the K63-linked ubiquitination of YY1 at the K339 site, subsequently leading to the proteasomal degradation of YY1. RNA-seq analysis and experimental validation have demonstrated that YY1 can suppress the induction of ferroptosis by regulating the transcription of downstream target genes under IRI conditions. Subsequent predictions and confirmations indicated that YY1 can transcriptionally upregulate the ferroptosis-related gene *SLC7A11*, thereby inhibiting IRI-induced ferroptosis and ameliorating liver injury. The role of iron in the process of hepatocellular IRI has attracted increasing attention. AXL receptor tyrosine kinase inhibits iron death (ferroptosis) through the PI3K/AKT signaling pathway to protect the liver [57]. Additionally, ubiquitination and deubiquitination play crucial roles in iron death and HIRI [58]. In future studies, we plan to further validate our findings by using conditional *NEDD4L*- or *YY1*-knockout mice. In summary, this study revealed that the E3 ubiquitin ligase NEDD4L facilitates the ubiquitination-mediated degradation of YY1, which in turn inhibits the transcription of the ferroptosis suppressor SLC7A11, consequently promoting the occurrence of IRI-associated ferroptosis and exacerbating liver injury ( Figure 7).

The scientific and clinical significance of this study lies in revealing the role of NEDD4L in promoting hepatocellular IRI by ubiquitinating and degrading YY1, thereby suppressing *SLC7A11* transcription, which exacerbates cellular ferroptosis. Elucidating this mechanism provides crucial insights for a better understanding of liver IRI and offers theoretical support for the development of new therapeutic strategies and drugs. From a clinical perspective, these findings may contribute to the design of targeted treatments for IRI, enhancing treatment efficacy and alleviating patient suffering. Future studies should involve validation of the expression and relationship of NEDD4L-YY1-SLC7A11 in human liver samples. 

Nonetheless, limitations of this study must be acknowledged, such as the fact that some experiments are still in the stage of *in vitro* validation and lack consistent verification in *in vivo* animal models, necessitating further in-depth research to establish the reliability of the conclusions. Moreover, a more comprehensive exploration of the roles of NEDD4L and YY1 in other physiological or pathological processes is needed to fully comprehend the integrated functions of these proteins in cell biology and disease progression. Future studies could explore the regulatory mechanism of cell ferroptosis via NEDD4L-mediated ubiquitination of YY1 and explore the molecular regulatory network of YY1 in IRI damage to identify additional potential therapeutic targets. Additionally, by integrating broader clinical data and patient samples, further validation of the research results can be undertaken to assess their clinical application potential, laying a more solid scientific foundation for personalized therapy targeting hepatic IRI. 

## Supporting information

294FigS1-3

294TableS1-4

## Supplementary Data

Supplementary data is available at *Acta Biochimica et Biophysica Sinica* online.
