# Supplementary material for: Modulation of ferroptosis via YY1-SLC7A11 axis in hepatic ischemia-reperfusion injury pathogenesis: Ferroptosis modulation in hepatic IRI
Source: Acta Biochim Biophys Sin (Shanghai). 2025 Jul 1;57(9):1391–405. doi: 10.3724/abbs.2025093 (PMC12536466; doi:10.3724/abbs.2025093)
Supplement: 294FigS1-3 [file 294FigS1-3.docx]

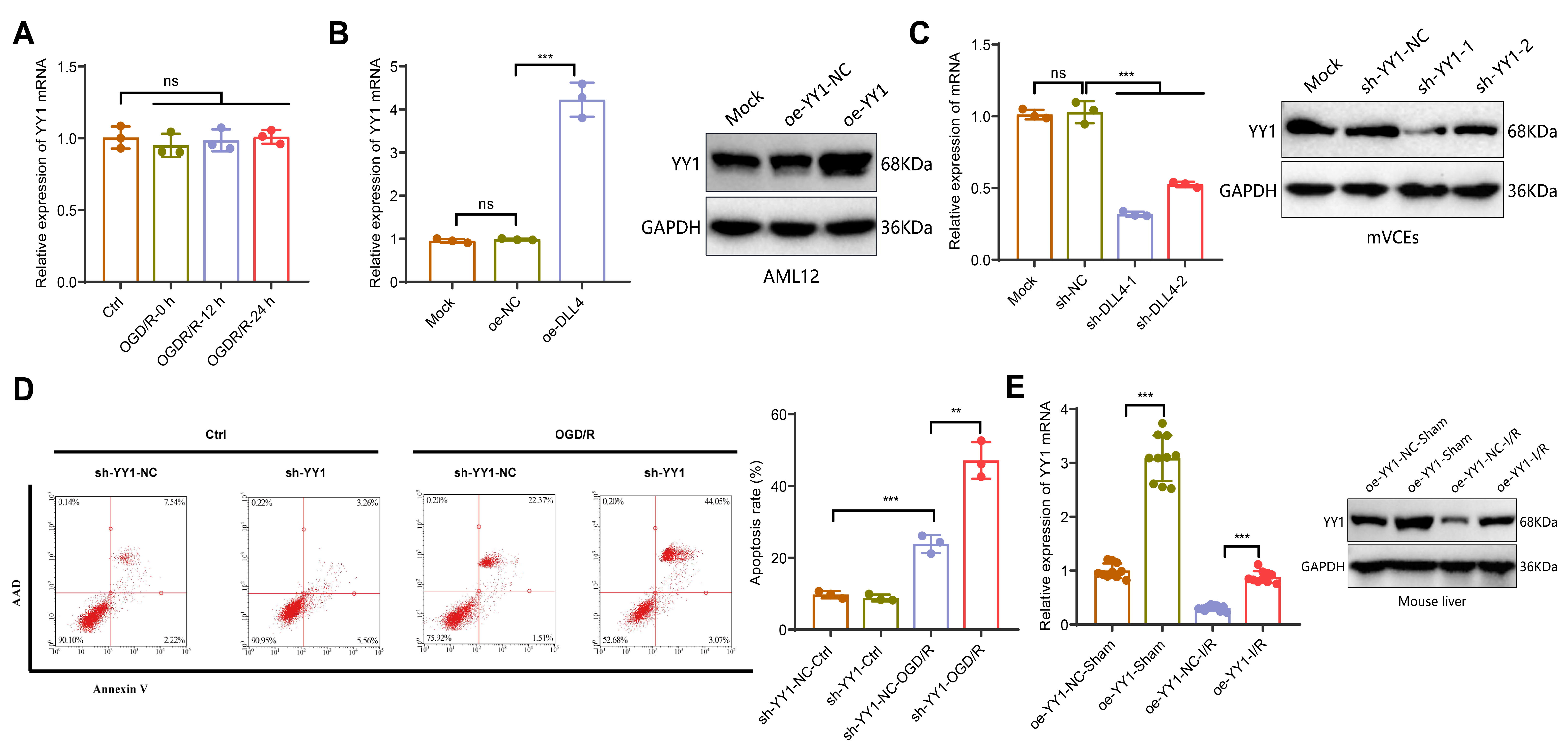


**Supplementary Figure S1. Validation of the effects of YY1 overexpression and knockdown on IRI** (A) Detection of AML12 YY1 mRNA expression after IRI-OGD/R treatment via RT-qPCR. (B,C) Verification of the effects of YY1 overexpression and knockdown in AML12 cells, as shown by RT-qPCR and western blot analysis; two different shRNA sequences were used for knockdown, with sh-YY1-1 chosen for subsequent experiments due to better knockdown efficiency. (D) Flow cytometry analysis of the impact of *YY1* knockdown on IRI-OGD/R-induced cell apoptosis in AML12 cells, with representative results on the left and a bar graph on the right. (E) Validation of YY1 overexpression in mouse liver tissues mediated by lentivirus, as shown by RT‒qPCR and western blot analysis (*n* = 10 per group); cell experiments were repeated 3 times. ^ns^*P* > 0.05, **P* < 0.05, ***P* < 0.01, ****P* < 0.001.


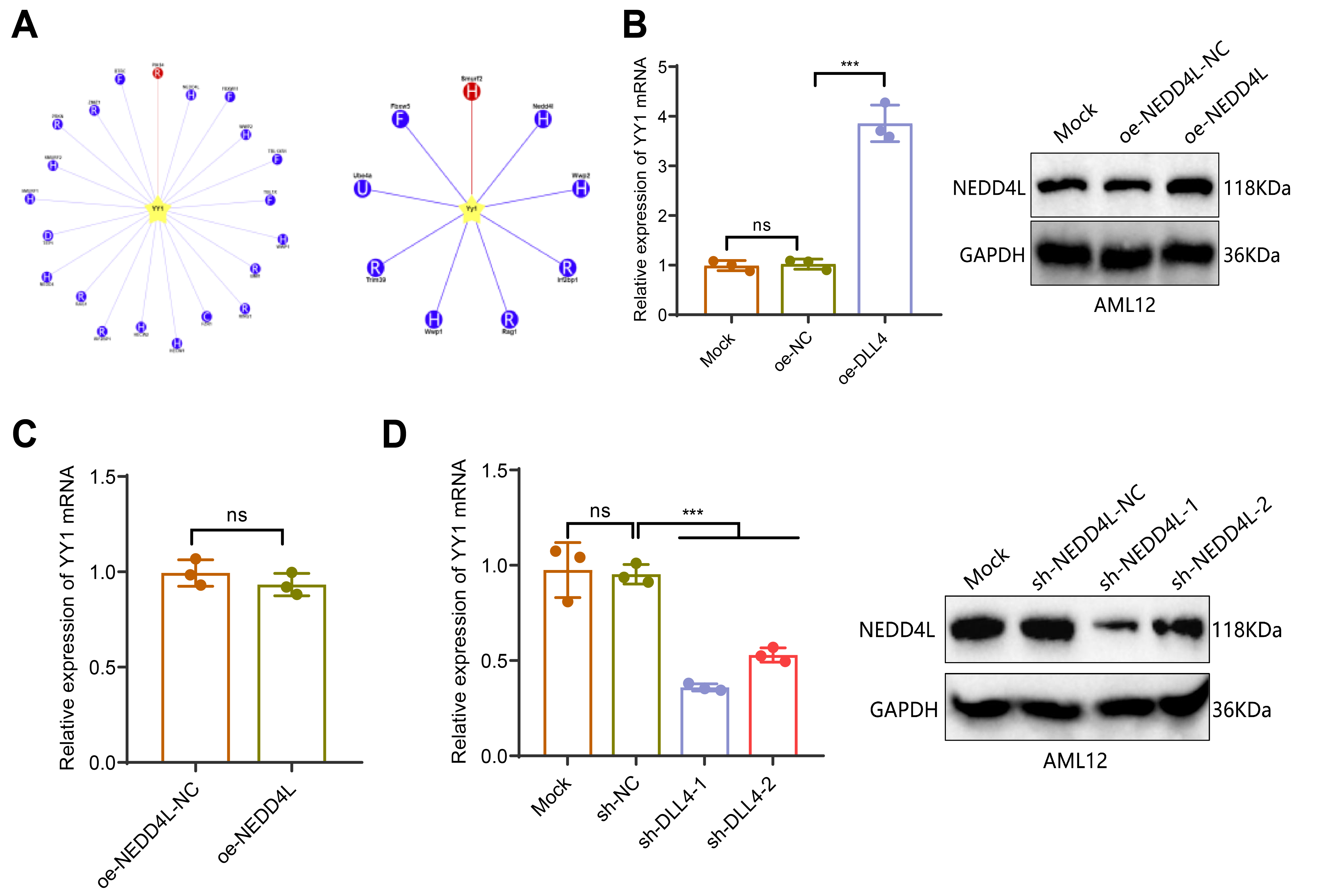


**Supplementary Figure S2. Screening of E3 ligases upstream of YY1 and validation of *NEDD4L* knockdown and overexpression** (A) Predicted E3 ligases of human (left) and mouse (right) YY1 obtained from the UbiBrowser 2.0 database. (B) Validation of NEDD4L overexpression in AML12 cells, shown by RT-qPCR and western blot analysis. (C) Detection of YY1 mRNA expression after NEDD4L overexpression via RT-qPCR. (D) Verification of *NEDD4L* knockdown effects in AML12 cells, demonstrated via RT-qPCR and western blot analysis; two different shRNA sequences were used for knockdown, with sh-NEDD4L-1 selected for subsequent experiments; cell experiments were repeated 3 times. ^ns^*P* > 0.05, **P* < 0.05, ***P* < 0.01, ****P* < 0.001.


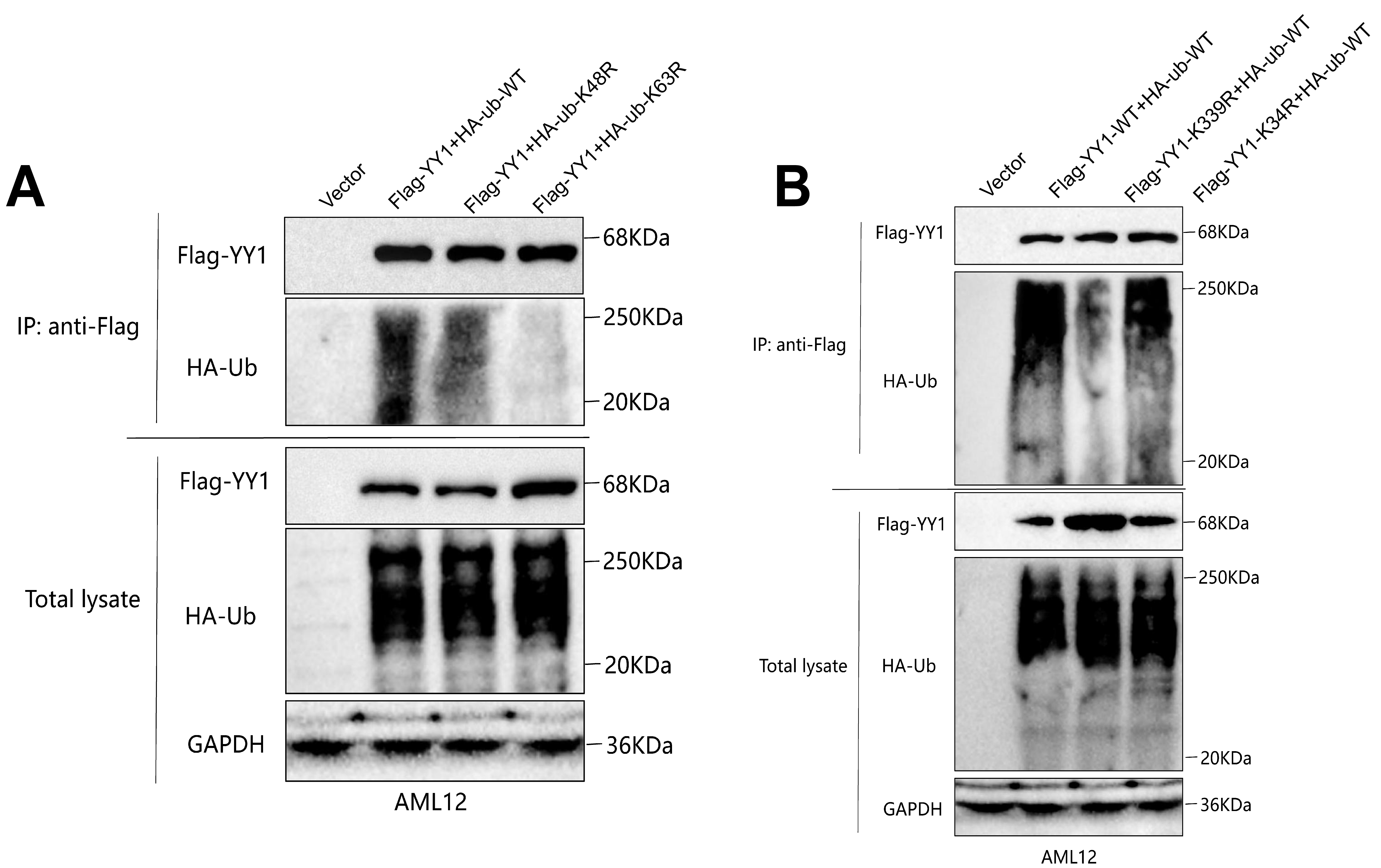


**Supplementary Figure S3. Validation of the main types of YY1 ubiquitination and ubiquitination sites in AML12 cells** (A) Co-IP and western blot analysis of the impact of Ub K48 and K63 site mutations on YY1 ubiquitination in AML12 cells. (B) Co-IP and western blot analysis of the effects of YY1 K339 and K341 site mutations on YY1 ubiquitination in AML12 cells. The experiments were repeated 3 times.
