# Supplementary material for: Modulation of ferroptosis via YY1-SLC7A11 axis in hepatic ischemia-reperfusion injury pathogenesis: Ferroptosis modulation in hepatic IRI
Source: Acta Biochim Biophys Sin (Shanghai). 2025 Jul 1;57(9):1391–405. doi: 10.3724/abbs.2025093 (PMC12536466; doi:10.3724/abbs.2025093)
Supplement: 294TableS1-4 [file 294TableS1-4.docx]

**Supplementary Table S1. Sequences of shRNAs used in the study**

| Name | shRNA sequence (5′→3′) |
| --- | --- |
| sh-NC | CCTAAGGTTAAGTCGCCCTCG |
| sh-YY1-1 (mouse) | CCCTAAGCAACTGGCAGAATT |
| sh-YY1-2 (mouse) | CGACGGTTGTAATAAGAAGTT |
| sh-NEDD4L-1 (mouse) | CCAGAGAGTTTAAGCAGAAAT |
| sh-NEDD4L-2 (mouse) | CGGAGGCACATTAGTGAAGAT |

**Supplementary Table S2. Sequences of primers used in ChIP-qPCR**

| Prediction site | Primer sequence (5′→3′) |
| --- | --- |
| P1 (mouse) | F: TTGGGCTCACGTATTGGATCA |
|  | R: TCCTAGGGTGGCACTTCCAT |
| P2 (mouse) | F: AGTTCCACATATCACACAGATGTA |
|  | R: GGGTGTATTCCCTGTGCAGT |
| P3 (mouse) | F: TGAGTTTCCTTCTCGCCTCAG |
|  | R: TTGCATGTCTACATGAACATGACTG |

F: forward; R: reverse.

**Supplementary Table S3. Information of antibodies used in western blot analysis**

| Target (host and reactivity) | Manufacturer | Cat. No. | Dilution |
| --- | --- | --- | --- |
| YY1 (rabbit anti-mouse) | Thermo Fisher Scientific | MA5-42708 | 1:500 |
| NEDD4L (rabbit anti-mouse) | Thermo Fisher Scientific | A302-513A | 1:2000 |
| SLC7A11 (rabbit anti-mouse) | Abcam | ab307601 | 1:1000 |
| Ub (rabbit anti-mouse) | Abcam | ab134953 | 1:1000 |
| Flag (rabbit monoclonal) | Abcam | ab205606 | 1:500 |
| HA (rabbit monoclonal) | Abcam | ab236632 | 1:1000 |
| GAPDH (mouse anti-mouse) | Abcam | ab8245 | 1:500 |
| β-actin (mouse anti-mouse) | Abcam | ab6276 | 1:5000 |

**Supplementary Table S4. Sequences of primers used in RT-qPCR**

| Gene (species) | Primer sequence (5′→3′) |
| --- | --- |
| YY1 (mouse) | F: GTGGTTGAAGAGCAGATCATTGG |
|  | R: TTGCTTAGGGTCTGAGAGGTC |
| NEDD4L (mouse) | F: GAGTCAAGGGGTTTTTGAGGTT |
|  | R: TGGGAAGCTGAGTCGTTGGA |
| SLC7A11 (mouse) | F: GGCACCGTCATCGGATCAG |
|  | R: CTCCACAGGCAGACCAGAAAA |
| IL-1β (mouse) | F: GAAATGCCACCTTTTGACAGTG |
|  | R: TGGATGCTCTCATCAGGACAG |
| TNF-α (mouse) | F: CTGAACTTCGGGGTGATCGG |
|  | R: GGCTTGTCACTCGAATTTTGAGA |
| MCP1 (mouse) | F: TAAAAACCTGGATCGGAACCAAA |
|  | R: GCATTAGCTTCAGATTTACGGGT |
| CXCL12 (mouse) | F: TGCATCAGTGACGGTAAACCA |
|  | R: CACAGTTTGGAGTGTTGAGGAT |
| CCL5 (mouse) | F: GCTGCTTTGCCTACCTCTCC |
|  | R: TCGAGTGACAAACACGACTGC |
| WWP2 (mouse) | F: TTTGAGAAGTCCCAGCTTACCC |
|  | R: CTCCAGACCTTCAGATCCAAATG |
| WWP1 (mouse) | F: GGCAGTCTCAGCGGAATCAAT |
|  | R: GGTCCATAGGGGTCATTTTCTG |
| IRF2BP1 (mouse) | F: GGTGCGTGAATTTCGAGGG |
|  | R: CTAGGTCCTTGGAAGTCGGGT |
| RAG1 (mouse) | F: ACCCGATGAAATTCAACACCC |
|  | R: CTGGAACTACTGGAGACTGTTCT |
| SMURF2 (mouse) | F: AAACAGTTGCTTGGGAAGTCA |
|  | R: TGCTCAACACAGAAGGTATGGT |
| TRIM39 (mouse) | F: ATGGCAGAGACAAGTCTGTTAGA |
|  | R: GGTAATGCACGCTTTGCAGAA |
| FBXW5 (mouse) | F: GAGTTCCGGCGGCTCTATG |
|  | R: AGATGGTCAGGTCGTTGTTCC |
| GAPDH (mouse) | F: AGGTCGGTGTGAACGGATTTG |
|  | R: TGTAGACCATGTAGTTGAGGTCA |

F: forward; R: reverse.
